# Combining stochastic models of air temperature and vapour pressure for the analysis of the bioclimatic comfort through the Humidex

**DOI:** 10.1038/s41598-020-68297-4

**Published:** 2020-07-09

**Authors:** Beniamino Sirangelo, Tommaso Caloiero, Roberto Coscarelli, Ennio Ferrari, Francesco Fusto

**Affiliations:** 1 Department of Environmental Engineering (DIAm), University of Calabria, Ponte P. Bucci, 87036 Rende, CS Italy; 2 National Research Council of Italy, Institute for Agriculture and Forest Systems in the Mediterranean (CNR-ISAFOM), Via Cavour 4/6, 87036 Rende, CS Italy; 3 National Research Council of Italy, Research Institute for Geo-Hydrological Protection (CNR-IRPI), Via Cavour 4/6, 87036 Rende, CS Italy; 4 Department of Computer Engineering, Modeling, Electronics, and Systems Science (DIMES), University of Calabria, Ponte P. Bucci, 87036 Rende, CS Italy; 5 Multi-Risk Functional Center, Regional Agency for Environmental Protection of Calabria, Viale degli Angioini 143, 88100 Catanzaro, Italy

**Keywords:** Hydrology, Natural hazards

## Abstract

Several studies evidenced the importance of the knowledge of the bioclimatic comfort for improving people’s quality of life. Temperature and relative humidity are the main variables related to climatic comfort/discomfort, influencing the environmental stress in the human body. In this study, a stochastic approach is proposed for characterizing the bioclimatic conditions through the Humidex values in six sites of Calabria (southern Italy), a region often hit by heat waves in summer months. The stochastic approach is essential, because the available time series of temperature and relative humidity are not long enough and present several missing values. The model allowed the characterization of sequences of extreme values of the Humidex. Results showed different behaviours between inner and coastal stations. For example, a sequence of 20 consecutive days with maximum daily Humidex values greater than 35 has a return period ranging from 10 to 20 years for the inner stations, while it exceeds 100 years for the coastal ones. The maximum yearly Humidex values for the inner stations have a larger range (40–50) than the coastal ones (38–45), reaching higher occurrence probabilities of serious danger conditions. Besides, the different influence of temperature and relative humidity on the Humidex behaviour has been evidenced.

## Introduction

Bioclimatic comfort is a cognitive process that mixes many stimuli influenced by physical, physiological, and psychological factors^1^. Previous researches evidenced the fundamental importance of the knowledge of the bioclimatic comfort for improving people’s quality of life. In fact, in the last years temperatures over 36 °C, especially in situations of heat waves, severely affected people’s health, mainly if children, elderly and chronically ill^2,3^.

An increase of severe heat waves in the current century has been forecasted in areas already affected by heat stress, such as the southern part of the European continent^4–6^ and the Mediterranean basin, which is expected to face particularly high impacts from global warming and climate change and to be most vulnerable to their deleterious effects^7^. The effects of heat can be various: increase of mortality^8^, working/exercise capacity^9,10^ and cognitive performance^11,12^.

For the analysis of bioclimatic comfort, in studies conducted at different spatial scales in various parts of the world (e.g. in America^13–16^, in Europe^17–19^, in Asia^20–22^ and in Oceania^23^) several parameters such as temperature, relative humidity of the air, wind and radiation have been analysed. In particular, given their influence on the environmental stress in the human body, temperature and the relative humidity of the air are the key basic parameters related to climatic comfort/discomfort^1^. In fact, as stated by Masterton and Richardson^24^, with different humidity conditions the same temperature can provide very different feelings to people. For this reason, in order to detect the effect of heat on human health, an index that combines the effects of temperature and humidity is therefore preferred^25^. In particular, to monitor and assess this effect, several indices have been developed. One of the most applied is the humidity index (Humidex) which is a relatively simple thermal comfort index based on air temperature and humidity^24^. It can be considered one of the most robust and effective comfort index, since its results are directly comparable with dry temperature in degrees Celsius and its values are associated with corresponding degrees of thermal comfort, rendering the index widely understandable. Moreover, the Humidex is easier to calculate than more complex indices (e.g. the Universal Thermal Climate Index or the Physiological Equivalent Temperature), as it requires only two input parameters (temperature and humidity) which are commonly measured by meteorological stations, because of their importance for every branch of atmospheric science^26^. In particular, the high accessibility of validation data for Humidex (e.g. in situ air temperature and humidity measurements, in contrast to mean radiant temperature usually needed by other models) has much to recommend it^25^. For these reasons, Humidex has been therefore widely used in several studies. For example, Błażejczyk and Twardosz^27^ studied the variability of bioclimatic conditions in Cracow (Poland) during the period of 1826–2006. Changes in extreme heat and extreme cold events represented by various Humidex and wind chill indices were analysed in Canada for the period 1953–2012 at 126 climatological stations by Mekis et al.^28^. The impact of the urban heat island on city residents and visitors was evaluated using the Humidex in Hradec Králové, Czech Republic^29^. Giannopoulou et al.^30^ performed an investigation on human thermal comfort in the Greater Athens area, during the period of June–August of 2009. The interactions between urbanization, heat stress, and climate change over the U.S. and southern Canada have been analysed by Oleson et al.^31^.

The study of the characteristics of the meteorological processes can be performed using the time series observed at weather stations. However, the analysis of the extreme behaviours of these processes is often unsuitable due to the small amount of data. In the last decades, the use of generation of synthetic data series obtained from the numerical implementation of stochastic models, with statistical properties similar to those of the real processes at the chosen time scale, has largely increased. Concerning the bioclimatic index Humidex, which depends only on temperature and relative humidity, two different numerical approaches can be applied for the stochastic analysis of the joint non-stationary time-series of these variables. Specifically, a first approach considers the diurnal fluctuation of the climatological processes, by assuming them as periodically correlated random processes with a 1-day period. Through a second approach, the meteorological events are thought as non-stationary random processes, to which the method of the inverse distribution functions can be applied for simulating non-Gaussian variables^32^. A similar procedure can also be employed to analyse previously deseasonalized variables in order to remove their cyclical variability^33,34^. A further difference between the two approaches concerns the numerical implementation of the models, which is faster for the models developed with the first approach, as the second one needs the joint time series of the two meteorological variables.

The aim of this paper is to characterize the Humidex distribution in a case study (Calabria region), and to assess the influence of temperature and humidity in determining the Humidex values. With these purposes, as the available time series are not long enough and with several missing values, a stochastic approach to deal with non-stationary random processes has been proposed for the characterization of the occurrence probability of sequences of extreme values of the Humidex, in a region which is usually hit by heat waves especially in summer periods. Through this approach, the parameters of the stochastic models of temperature and relative humidity can be jointly estimated from the observed time series, and then synthetic values of Humidex can be obtained from the generated time series.

## Materials and methods

### The Humidex

The Humidex (*H*) is an index which combines temperature and humidity to better describe the effect of heat on living organisms. The index is expressed by the empirical equation:

1 $$ H = T + \frac{5}{9}\left( {e - 10} \right), $$

where *T* is the air temperature (*°*C), and *e* is the partial vapour pressure (hPa)^24^. As this last variable is not easily available, it can be evaluated by using the relative humidity, *U*_*r*_ (%), and the saturation vapour pressure, *e*_*sat*_ (hPa), through the relationship:

2 $$e = \frac{U_{r}\cdot e_{sat}}{100},$$

where *e*_*sat*_ depends on the air temperature alone, and can be calculated through the Tetens’ formula^35^:

3 $$ e_{sat} = 6.112 \cdot 10^{{\frac{7.5 \cdot T}{{T + 237.7}}}} . $$

The Humidex has no specific measurement unit, anyway it can be associated to the same unit of the temperature (*°*C), though it is not a physical variable. As a result, the temperature perceived by human body can be easily found by using the observed values of temperature and relative humidity in Eqs. (1)–(3), and detecting the discomfort level corresponding to the Humidex value (Table 1).

**Table 1:** Discomfort levels for different classes of the Humidex index^35^.

Humidex	State
*H* < 29	Discomfort perceived by a few people
30 < *H* < 34	More or less significant malaise
35 < *H* < 39	Quite intense malaise. Caution. Limit some heavy physical activities
40 < *H* < 45	Sense of general malaise. Danger. Avoid efforts
46 < *H* < 53	Serious danger. Suspend physical activities
*H* > 54	Impending heatstroke (danger of death)

### Stochastic models

The stochastic approach here proposed analyses the couple temperature—partial pressure of water vapour at the daily scale, whose data have been evaluated starting from an hourly dataset. In particular, given the higher influence of the temperature in the evaluation of the Humidex, for each day the maximum hourly temperature value and the corresponding humidity data have been considered as daily value.

Since the partial pressure of the water vapour is always positive, its natural logarithm has been considered, *Le*(*t*) = ln(*e*(*t*)), in order to model couples of *T*-ln(*e*) values whose elements can be more easily treated through the linear stochastic modelling. In particular, the sequences of daily temperature, *T*(*i*), and partial pressure of water vapour, *Le*(*i*), of the i-day starting from a generic point (i = 0,1,2,…), can be generally recognized as a realization of discrete parameter stochastic processes with cyclostationarity features in a period equal to 1 year.

#### Deseasonalisation and Gaussianization procedures

To deal with the seasonal features of the variables, each of the *T*(*i*) and the *Le*(*i*) processes can be separately reduced to a weakly stationary standardized process, respectively termed as *X*(*i*) and *Y*(*i*), through the transformation:

4 $$ X(i) = \frac{{T(i) - \mu_{T} (i)}}{{\sigma_{T} (i)}}, $$

5 $$ Y(i) = \frac{{Le(i) - \mu_{Le} (i)}}{{\sigma_{Le} (i)}}, $$

where $\mu_{T} (i)$ and $\sigma_{T} (i)$ are the mean and the standard deviation functions of the *T*(*i*) process, $\mu_{Le} (i)$ and $\sigma_{Le} (i)$ are the analogous functions of the *Le*(*i*) process. In particular, the functions $\mu_{T} (i)$, $\sigma_{T}^{2} (i)$, $\mu_{Le} (i)$ and $\sigma_{Le}^{2} (i)$ can be obtained by means of truncated expansion Fourier series that are composed by one or more harmonics, allowing for different values of the Fourier coefficients, generally estimated through the least squares method. In order to make the better choice about the number of harmonics, also taking into account the parsimony principle, the behaviour of the different Fourier series have to be compared at annual scale to the correspondent observed mean daily values of $\mu_{T} (i)$, $\sigma_{T}^{2} (i)$, $\mu_{Le} (i)$ and $\sigma_{Le}^{2} (i)$. This can be performed through a simple test, which however requires independent data. To this aim, subsamples from the *X*(*i*) and *Y*(*i*) series can be extracted for each couple of number of harmonics hypothesized for the mean and the standard deviation functions of *T*(*i*) and *Le*(*i*) (see Eqs. 4 and 5). Finally, by splitting the subsamples into separate classes with distinct mean and variance values, the hypotheses of equality of the means and the variances of each class can be tested^33^.

The sample values of the random variables *X*(*i*) and *Y*(*i*) have a null mean value and a unit variance, but generally climatic variables, once deseasonalized, show skewness and kurtosis coefficients significantly different from the theoretical values expected for a normal variable^36^. On the other side, the Gaussianization step is needed for developing a coherent linear stochastic model. To cope with such problems, the deseasonalized variables *X*(*i*) and *Y*(*i*) can be converted into standardized normal variables, *U*(*i*) and *V*(*i*), respectively, by means of the transformation functions introduced by Johnson^37^, whose general equation applied to the random variables *X*(*i*) and *Y*(*i*) provides the following relationships:

6 $$ U = \eta_{u} + \theta_{u} \cdot \ln \left[ {f_{X} \left( {x;\alpha_{u} ,\beta_{u} } \right)} \right], $$

7 $$ V = \eta_{v} + \theta_{v} \cdot \ln \left[ {f_{Y} \left( {y;\alpha_{v} ,\beta_{v} } \right)} \right], $$

where − ∞ < *η*_*u,*_*η*_*v*_ < + ∞, *θ*_*u*_*,θ*_*v*_ > 0, − ∞ < *α*_*u*_*,α*_*v*_ <  + ∞, and *β*_*u*_*,β*_*v*_ > 0 are the parameters of the transformations. The functions $f_{X} \left( {x;\alpha_{u} ,\beta_{u} } \right)$ and $f_{Y} \left( {y;\alpha_{v} ,\beta_{v} } \right)$ can assume one of the forms known as unbounded and bounded Johnson transformations, and log-normal law with 3 parameters, depending on the sample values of the skewness (*g*_*1,X*_ and *g*_*1,Y*_) and the kurtosis coefficients (*g*_*2,X*_ and *g*_*2,Y*_). For details on the statistical procedure used for the estimation of the parameters, see Sirangelo et al.^33^.

#### Analysis of the correlative structure

Generally, a marked persistence can still characterize the correlative structures of the sample data series *U*(*i*) and *V*(*i*) obtained from the Johnson transformations. These structures can be explained through *FARIMA* (fractionally differenced autoregressive integrated moving average) models^38–40^, that, with reference only to the variable *U*, can be described by the following expression:

8 $$ \Phi_{{u,p_{u} }} (B)(1 - B)^{{d_{u} }} u(i) = \Psi_{{u,q_{u} }} (B)\varepsilon_{u} (i), $$

where *B* is the backward operator, $\Phi_{{u,p_{u} }} \left( B \right)$ is the *p*-order polynomial of the autoregressive component of the *u*(*i*) process, $\Psi_{{u,q_{u} }} \left( B \right)$ is the *q*-order polynomial of its mean average component, $\varepsilon_{u} \left( i \right)$ is a sequence of i.i.d. random variables with mean zero and standard deviation $\sigma_{{\varepsilon_{u} }}$, and *d*_*u*_ is its fractional order of differentiation. The *FARIMA*(*p*_*u*_*, d*_*u*_*, q*_*u*_) model adopted for the *u*(*i*) process embodies a fractional filter *d*_*u*_ and an *ARMA*(*p*_*u*_*, q*_*u*_) process, which is assumed to describe the intermediate process:

9 $$u^{*}(i) = (1 - B)^{{d_{u} }} u(i).$$

For each assigned value of the parameter *d*_*u*_, the sample *u*(*i*) can be transformed into a sample u^*^(*i*), which represents a realization of the *ARMA*(*p*_*u*_*, q*_*u*_) process, that can be expressed as:

10 $$u^{*}(i) = \sum\limits_{{k_{p} = 1}}^{{p_{u} }} {\phi_{{u,k_{p} }} } u^{*}(i - k_{p} ) + \sum\limits_{{k_{q} = 0}}^{{q_{u} }} {\psi_{{u,k_{q} }} } \varepsilon_{u} (i - k_{q} ).$$

The Eqs. (8)–(10) can be implemented also for the *v*(*i*) process by adopting analogous parameters ($\Phi_{{v,p_{v} }} \left( B \right)$, $\Psi_{{v,q_{v} }} \left( B \right)$, *d*_*v*_, $\varepsilon_{v} \left( i \right)$ and $\sigma_{{\varepsilon_{v} }}$).

A procedure for the contextual estimation of the parameters of the two processes *u*(*i*) and *v*(*i*) can now be performed. By setting a value for each fractional filter of the FARIMA models, *d*_*u*_ and *d*_*v*_, two intermediate realizations u^*^(*i*) and v^*^(*i*) of the processes ARMA(*p*_*u*_, *q*_*u*_) and ARMA(*p*_*v*_, *q*_*v*_), respectively, can be separately obtained [e.g., Eq. (9) for the *u*(*i*) process]. Hence, the parameters *ϕ*_*u*,1_,…,*ϕ*_*u,pu*_,*ψ*_*u*,1_,…,*ψ*_*u,qu*_ and *ϕ*_*v*,1_,…,*ϕ*_*v,pv*_,*ψ*_*v*,1_,…,*ψ*_*v,qv*_ of the two ARMA processes can be distinctly evaluated by prefixing the values of the orders (*p*_*u*_, *q*_*u*_) and (*p*_*v*_, *q*_*v*_) of the autoregressive and mean average components [e.g., Eq. (10) for the u^*^(*i*) process]. Ultimately, varying in a joint way the parameters (*p*_*u*_, *q*_*u*_, *d*_*u*_) for the *u*(*i*) process and (*p*_*v*_, *q*_*v*_, *d*_*v*_) for the *v*(*i*) process, a trial and error technique allows to obtain a correlation value between the errors *ε*_*u*_ and *ε*_*v*_ of the two ARMA models, $\hat{\rho }_{{\varepsilon_{U*,} \varepsilon_{V*} }}$, reproducing the sample cross-correlation value observed between the variables *U* and *V*, *r*_*U,V*_.

### Study area and data

Located at the toe of the Italian peninsula, Calabria has a typically Mediterranean climate. It features sharp contrasts due to both its position within the Mediterranean Sea and its orography. Specifically, warm air currents coming from Africa affect the Ionian side, leading to high temperatures, and to short and heavy precipitation. The Tyrrhenian side, instead, is affected by western air currents, which cause milder temperatures and more intense precipitations if compared to the Ionian side. Cold and snowy winters, and fresh summers with some precipitation, are typical of the inner areas of the region^41,42^.

**Figure 1:**
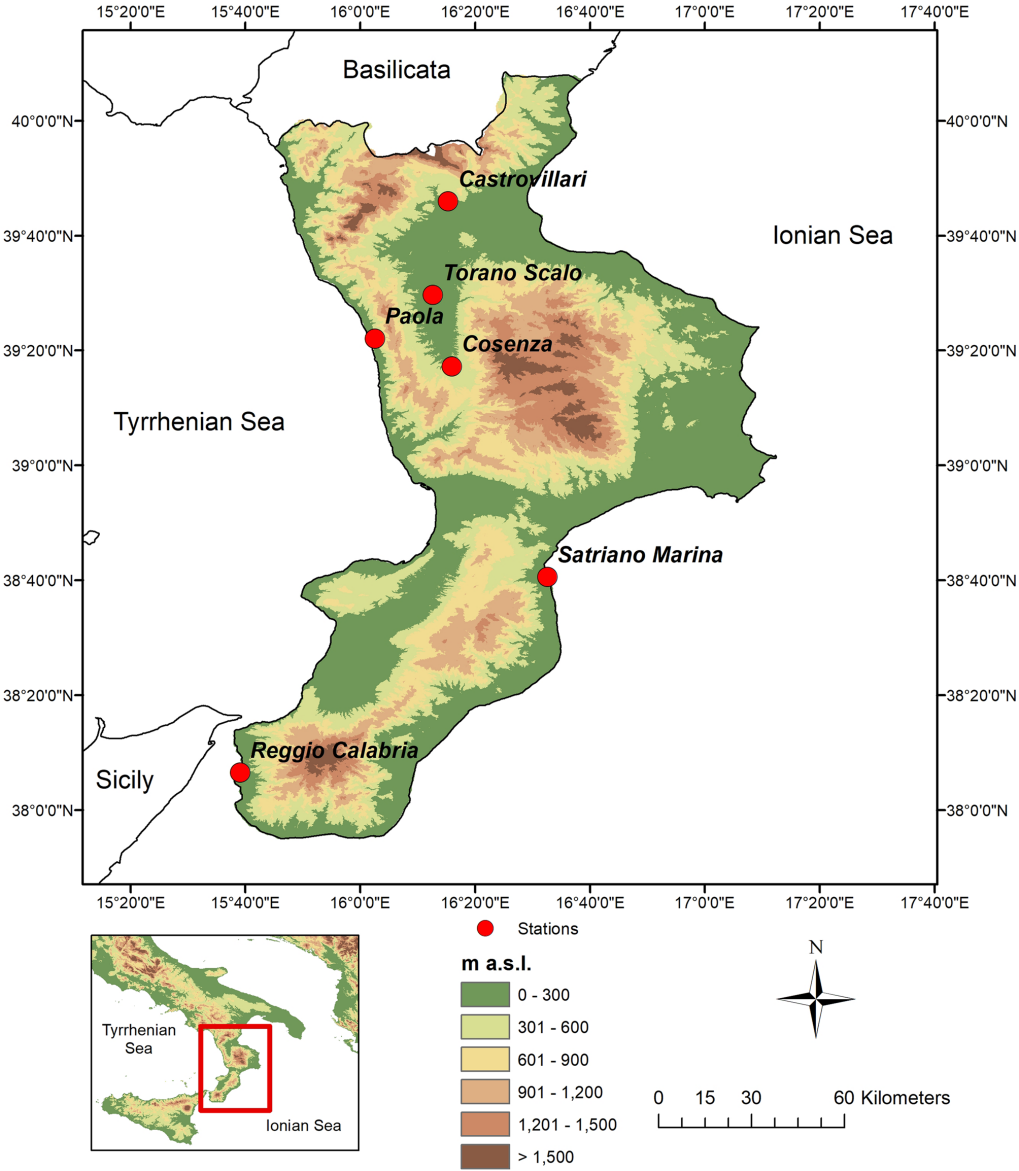
Localization of the stations on a Digital Elevation Model (DEM) of the Calabria region (created with Arcgis 10.4.1, https://desktop.arcgis.com/en/).

In this paper, the original database is composed by a set of hourly time series of air temperatures, *T* (°C), and relative humidity, *Ur* (%). In particular, six stations managed by the Multi-Risk Functional Centre of the Regional Agency for Environment Protection (Fig. 1) have been considered. Through a preliminary exploratory analysis focused on the data quality, the hourly values which lead relative humidity to jump from less than 75–100% or viceversa have been detected and consequently discarded from the original data. The main features of the databases are presented in Table 2.

**Table 2:** Main features of the selected series.

Station	First day	Last day	N. of data	Missing data (%)
Castrovillari	04/05/2001	13/02/2019	6,494	2.7
Cosenza	01/01/2001	31/12/2018	6,573	1.1
Paola	03/12/2000	13/02/2019	6,646	1.6
Reggio Calabria	03/04/2001	13/02/2019	6,525	3.1
Satriano Marina	03/12/2000	13/02/2019	6,646	1.0
Torano Scalo	03/12/2000	13/02/2019	6,646	0.5

## Results

### Parameter estimation

**Figure 2:**
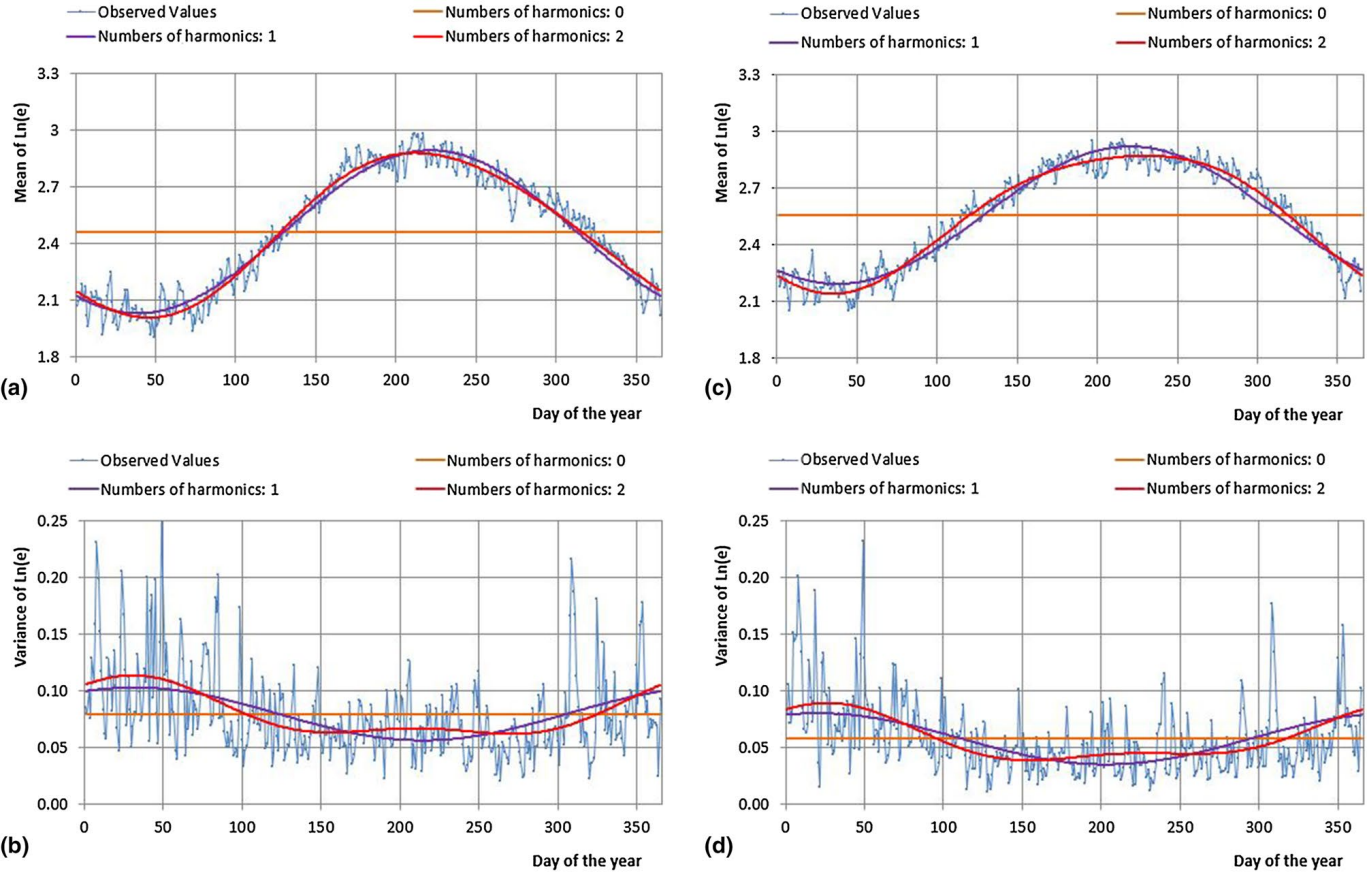
Comparison between observed values and Fourier series expansions (with 0, 1 and 2 harmonics) for the mean (**a**, **c**) and the variance functions (**b**, **d**) of *T* and *Le*, respectively, for the Cosenza and Torano Scalo stations.

First, the functions $\mu_{T} (i)$, $\sigma_{T}^{2} (i)$, $\mu_{Le} (i)$ and $\sigma_{Le}^{2} (i)$ have been defined by means of the development of truncated Fourier series. In order to verify the adaptation of these functions, characterized by different numbers of harmonics, Fig. 2 shows the comparison at annual scale of the observed mean daily values and the corresponding functions of $\mu_{T} (i)$ and $\sigma_{T}^{2} (i)$ for the Cosenza station, and of $\mu_{Le} (i)$ and $\sigma_{Le}^{2} (i)$ to for the Torano Scalo station.

Due to parsimony criteria, for each function, the minimum number of harmonics to be used in the truncated Fourier series has been evaluated as the one which allows not to reject the null hypothesis with a significance level of 5%. As an example, with reference to the Cosenza station, Table 3 shows some trials of the test used for the identification of the minimum number of harmonics of the Fourier expansions. As can be seen, for the functions $\sigma_{T}^{2} (i)$, $\mu_{Le} (i)$ and $\sigma_{Le}^{2} (i)$ the hypothesis of one harmonic cannot be rejected for all the classes. As regards the function $\mu_{T} (i)$, the hypothesis of one harmonic is rejected for the 4th and the 7th classes, thus a further harmonic is needed.

**Table 3:** Results of the rejection tests for different number of harmonics for the functions $\mu_{T} (i)$, $\sigma_{T}^{2} (i)$, $\mu_{Le} (i)$ and $\sigma_{Le}^{2} (i)$ of the Cosenza gauge.

	Classes									
	1	2	3	4	5	6	7	8	9	10
**Percentiles of** ${\user2{\mu }}_{\user2{T}} (\user2{i})$										
*m*_*T*,0.025_	11.66	12.57	16.73	21.45	27.37	30.21	29.54	23.80	18.37	12.75
*m*_*T*,0.975_	13.17	14.13	18.31	22.97	28.98	31.50	30.92	25.27	19.98	14.21
**0 Harmonics**										
*μ*_*T*_(*i*)	21.28	21.28	21.28	21.28	21.28	21.28	21.28	21.28	21.28	21.28
*H*_0_ rejected	Y	Y	Y	Y	Y	Y	Y	Y	Y	Y
**1 Harmonic**										
*μ*_*T*_(*i*)	11.80	13.08	17.48	23.32	28.37	30.71	29.43	25.02	19.19	14.13
*H*_0_ rejected	N	N	N	Y	N	N	Y	N	N	N
**2 Harmonics**										
*μ*_*T*_(*i*)	12.25	13.81	17.49	22.60	27.93	31.16	30.15	25.03	18.47	13.69
*H*_0_ rejected	N	N	N	N	N	N	N	N	N	N
**Percentiles of** $\user2{\mu}_{\user2{{Le}}} {\user2{(i)}}$										
*m*_*ln*(*e*),0.025_	1.97	1.96	2.11	2.40	2.66	2.82	2.79	2.64	2.48	2.17
*m*_*ln*(*e*),0.975_	2.11	2.11	2.23	2.50	2.77	2.91	2.90	2.74	2.60	2.30
**0 Harmonics**										
*μ*_*ln*(*e*)_(*i*)	2.47	2.47	2.47	2.47	2.47	2.47	2.47	2.47	2.47	2.47
*H*_0_ rejected	Y	Y	Y	N	Y	Y	Y	Y	Y	Y
**1 Harmonic**										
*μ*_*ln*(*e*)_(*i*)	2.06	2.05	2.20	2.45	2.70	2.87	2.88	2.73	2.49	2.23
*H*_0_ rejected	N	N	N	N	N	N	N	N	N	N
**Percentiles of** ${\user2{\sigma}}_{\user2{{T}}}^{\user2{{2}}} {\user2{(i)}}$										
*s*^*2*^_*T*,0.025_	10.16	10.89	11.76	10.64	11.82	7.63	8.32	9.60	9.81	10.21
*s*^*2*^_*T*,0.975_	19.09	20.45	22.09	20.00	22.21	14.34	15.63	18.04	18.43	19.19
**0 Harmonics**										
*σ*^*2*^_*T*_(*i*)	11.61	11.61	11.61	11.61	11.61	11.61	11.61	11.61	11.61	11.61
*H*_0_ rejected	N	N	Y	N	Y	N	N	N	N	N
**1 Harmonic**										
*σ*^*2*^_*T*_(*i*)	12.13	13.10	13.50	13.18	12.25	11.08	10.11	9.72	10.04	10.96
*H*_0_ rejected	N	N	N	N	N	N	N	N	N	N
**Percentiles of **${\user2{\sigma}}_{\user2{{Le}}}^{\user2{{2}}} {\user2{(i)}}$										
*s*^*2*^_*ln*(*e*),0.025_	0.093	0.096	0.059	0.052	0.058	0.041	0.055	0.051	0.068	0.079
*s*^*2*^_*ln*(*e*),0.975_	0.174	0.179	0.112	0.098	0.110	0.076	0.104	0.096	0.127	0.149
**0 Harmonics**										
*σ*^*2*^_*ln*(*e*)_(*i*)	0.080	0.080	0.080	0.080	0.080	0.080	0.080	0.080	0.080	0.080
*H*_0_ rejected	Y	Y	N	N	N	Y	N	N	N	N
**1 Harmonic**										
*σ*^2^_*ln*(*e*)_(*i*)	0.103	0.101	0.092	0.078	0.065	0.057	0.058	0.068	0.082	0.095
*H*_0_ rejected	N	N	N	N	N	N	N	N	N	N

Similarly to the Cosenza station, the number of the harmonics has been estimated for each station. The results, indicated in Table 4, show that in order to remove the periodicity in the mean function $\mu_{T} (i)$, two harmonics are needed for all the stations. For the functions $\sigma_{T}^{2} (i)$, $\mu_{Le} (i)$ and $\sigma_{Le}^{2} (i)$ the number of harmonics varies between 1 and 2. In particular, 2 harmonics are required for Paola and Torano Scalo as regards $\sigma_{T}^{2} (i)$, for Castrovillari, Reggio Calabria and Torano Scalo with respect to $\mu_{Le} (i)$, and for Castrovillari when the function $\sigma_{Le}^{2} (i)$ is considered.

**Table 4:** Number of harmonics chosen for the functions $\mu_{T} (i)$, $\sigma_{T}^{2} (i)$, $\mu_{Le} (i)$ and $\sigma_{Le}^{2} (i)$ of all the stations.

Station	$\mu_{T} (i)$	$\sigma_{T}^{2} (i)$	$\mu_{Le} (i)$	$\sigma_{Le}^{2} (i)$
Castrovillari	2	1	2	2
Cosenza	2	1	1	1
Paola	2	2	1	1
Reggio Calabria	2	1	2	1
Satriano Marina	2	1	1	1
Torano Scalo	2	2	2	1

The gaussianisation procedure, performed through the Johnson transformation, has been applied to the deseasonalised data series *X*(*i*) and *Y*(*i*)*.* The results show that the Gaussian functions *U*(*i*) and *V*(*i*) required the use of the unbounded version for all the stations, with the exception of the *U* function for Torano Scalo, which required the bounded version.

**Figure 3:**
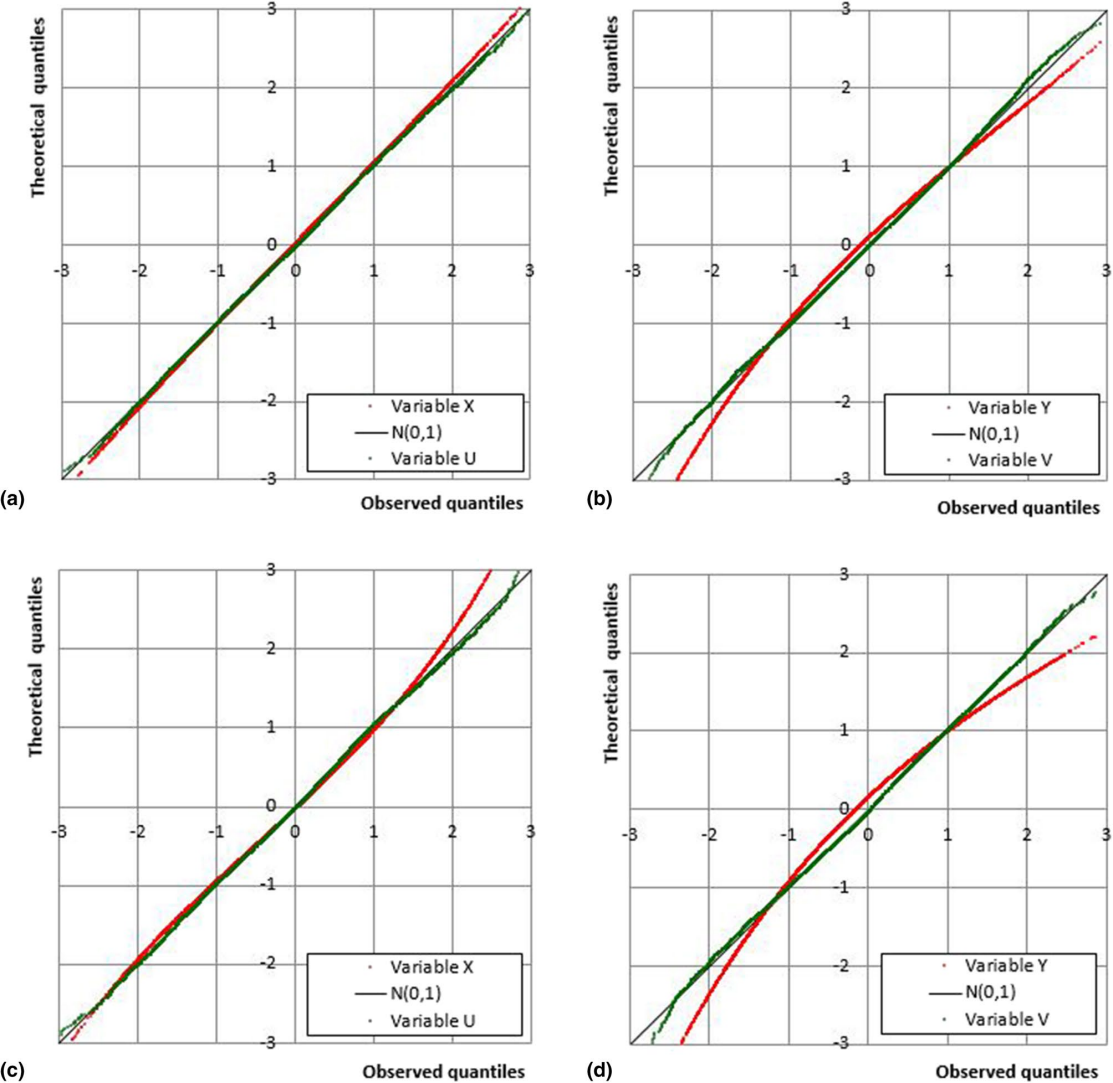
Q–Q plot of *X* vs *U*, and of *Y* vs *V* for the Cosenza (**a**, **b**) and Paola (**c**, **d**) gauges.

Figure 3 shows the comparisons between theoretical and observed quantiles of the standardized Gaussian laws for the variables *X* and *U*, and *Y* and *V* evaluated for the Cosenza and Paola stations. Generally, better performances appear for the function *U*, while for *V* some discordances between theoretical and sample values have been observed, especially for the extreme ones.

**Figure 4:**
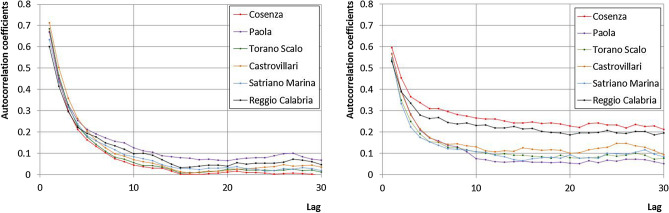
Autocorrelograms of the *U* (left) and *V* (right) variables for all the considered stations.

Figure 4 presents the autocorrelograms of *U* and *V* evaluated for each station considering a maximum lag value equals to 30. For the variable *U,* a similar behaviour for all the station has been detected. In particular, the autocorrelation coefficients rapidly decrease, reaching values lower than 0.1 after 10 days. For the variable *V* the persistency is stronger, especially for the Cosenza and Reggio Calabria stations, for which the autocorrelation coefficients are still about 0.2 after 30 days. The autocorrelation coefficients of the remaining stations decrease faster, achieving a value of about 0.1 just after 10 days.

**Figure 5:**
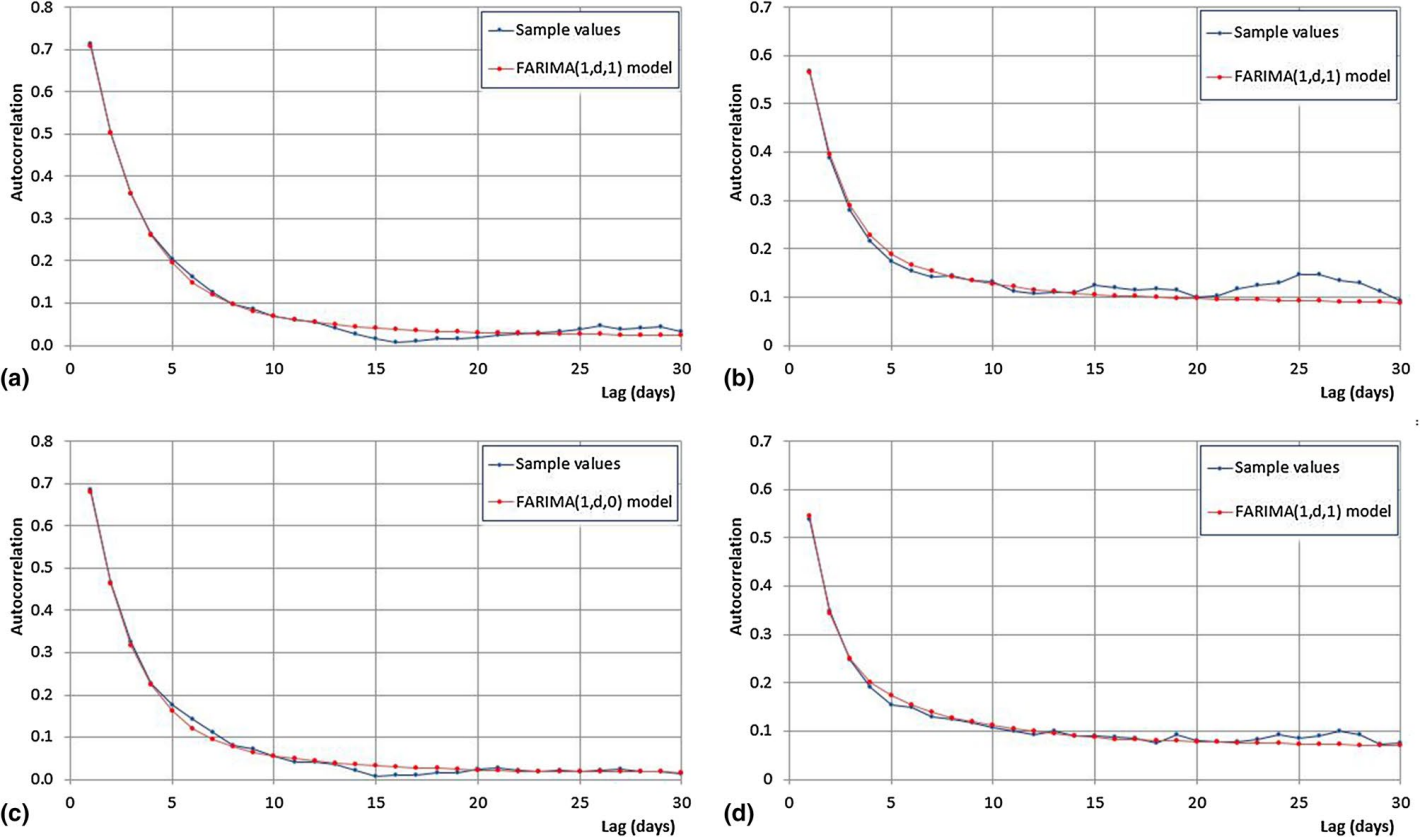
Comparison between theoretical and observed autocorrelograms of the variables *U* (**a**, **c**) and *V* (**b**, **d**) respectively, for the Castrovillari and Torano Scalo stations.

The identification of the *FARIMA*(*p*,* d*,* q*) process allows to describe the correlative structure of the Gaussian series. The results of the process are summarised in Table 5, in which the *FARIMA* parameters of both *U* and *V* are shown for the 6 stations. A *FARIMA*(1, $\hat{d}$, 0) model was identified in 3 out of 12 cases (Cosenza, Paola, and Torano Scalo for the variable *U*), while for the other 9 cases a *FARIMA*(1, $\hat{d}$, 1) model was detected. Figure 5 shows the comparison between the experimental and the theoretical correlograms for the Castrovillari and the Torano Scalo stations for both the functions. Results clearly evidence that the *FARIMA* model well reproduces the long-term memory.

**Table 5:** Estimated values of the parameters of the *FARIMA* processes applied to all the stations.

Station	Variable	*FARIMA*	$\hat{d}$	$\hat{\phi }_{1}$	$\hat{\psi }_{1}$	$\hat{\sigma }_{\varepsilon }$	$\hat{\rho }_{{\varepsilon_{{U_{*} }} ,\varepsilon_{{V_{*} }} }}$	$r_{U,V}$
Castrovillari	*U*	$\left( {1,d,1} \right)$	0.093	0.631	− 0.019	0.706	0.180	0.173
	*V*	$\left( {1,d,1} \right)$	0.212	0.483	− 0.220	0.827		
Cosenza	*U*	$\left( {1,d,0} \right)$	0.084	0.588	–	0.741	0.202	0.183
	*V*	$\left( {1,d,1} \right)$	0.381	0.456	− 0.466	0.792		
Paola	*U*	$\left( {1,d,0} \right)$	0.205	0.442	–	0.741	0.374	0.371
	*V*	$\left( {1,d,1} \right)$	0.181	0.447	− 0.090	0.807		
Reggio Calabria	*U*	$\left( {1,d,1} \right)$	0.186	0.522	− 0.175	0.791	0.140	0.136
	*V*	$\left( {1,d,1} \right)$	0.340	0.060	− 0.047	0.834		
Satriano Marina	*U*	$\left( {1,d,1} \right)$	0.126	0.594	− 0.140	0.771	0.202	0.199
	*V*	$\left( {1,d,1} \right)$	0.205	0.367	− 0.096	0.839		
Torano Scalo	*U*	$\left( {1,d,0} \right)$	0.087	0.601	–	0.728	0.137	0.132
	*V*	$\left( {1,d,1} \right)$	0.221	0.286	− 0.014	0.833

### Application of the model

**Figure 6:**
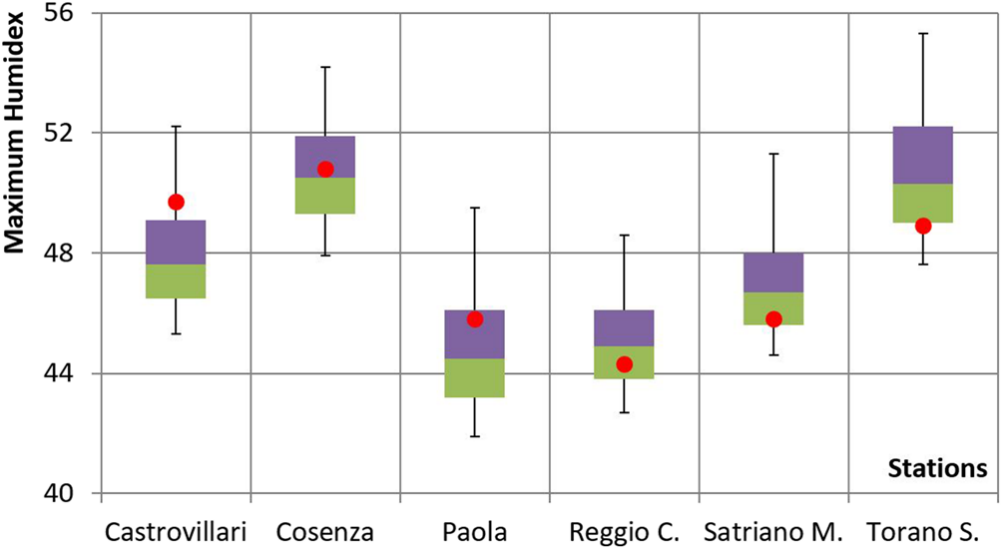
Comparison between the average values of the sample maximum annual Humidex (red points) and the maximum annual values of Humidex obtained from the synthetic series (box-plots). (Bottom and top of the box: 25th and 75th percentiles. Band inside the box: 50th percentiles. Ends of the whiskers: 5th and 95th percentiles. Green colour: values below the median. Violet colour: values above the median).

By means of the proposed stochastic model and the estimation of its parameters for each station, 10^5^ synthetic annual series of daily values of *T* and *Ur* using a Monte Carlo procedure have been generated, also taking into account leap years. The paired values of these variables have been used to evaluate a corresponding number of annual series of daily Humidex values. In Fig. 6, the average values of the maximum annual Humidex evaluated from the observed data for all the stations are superimposed to the box-plots of the maximum annual values of Humidex obtained from the synthetic series. As a result, all the sample average values fall within the range of the percentiles 25–75%, with the exception of the value observed for the Castrovillari station, which is higher than the 75% percentile.

**Figure 7:**
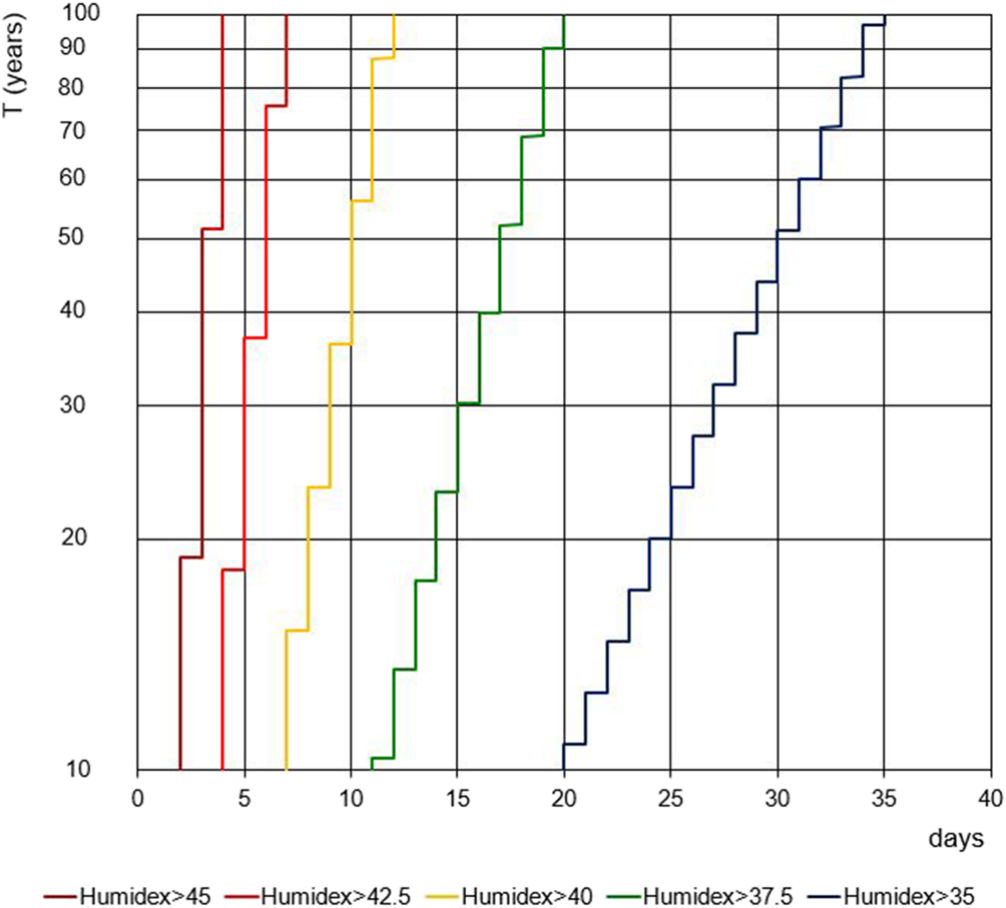
Annual maximum values of the number of consecutive days with maximum daily value of Humidex greater than specified thresholds for the Cosenza station.

Figure 7 shows for the Cosenza station the return period of the maximum yearly values of consecutive days with maximum daily Humidex values greater than prefixed thresholds. Obviously, for a fixed return period, the sequences of consecutive days shorten for increasing threshold values. In particular, for a return period of 10 years the sequences vary between 2 and 20 days for a Humidex threshold of 45 and 35, respectively. At the same time, considering a return period of 100 years, the sequences vary from 4 to 35 days for a Humidex threshold of 45 and 35, respectively.

**Figure 8:**
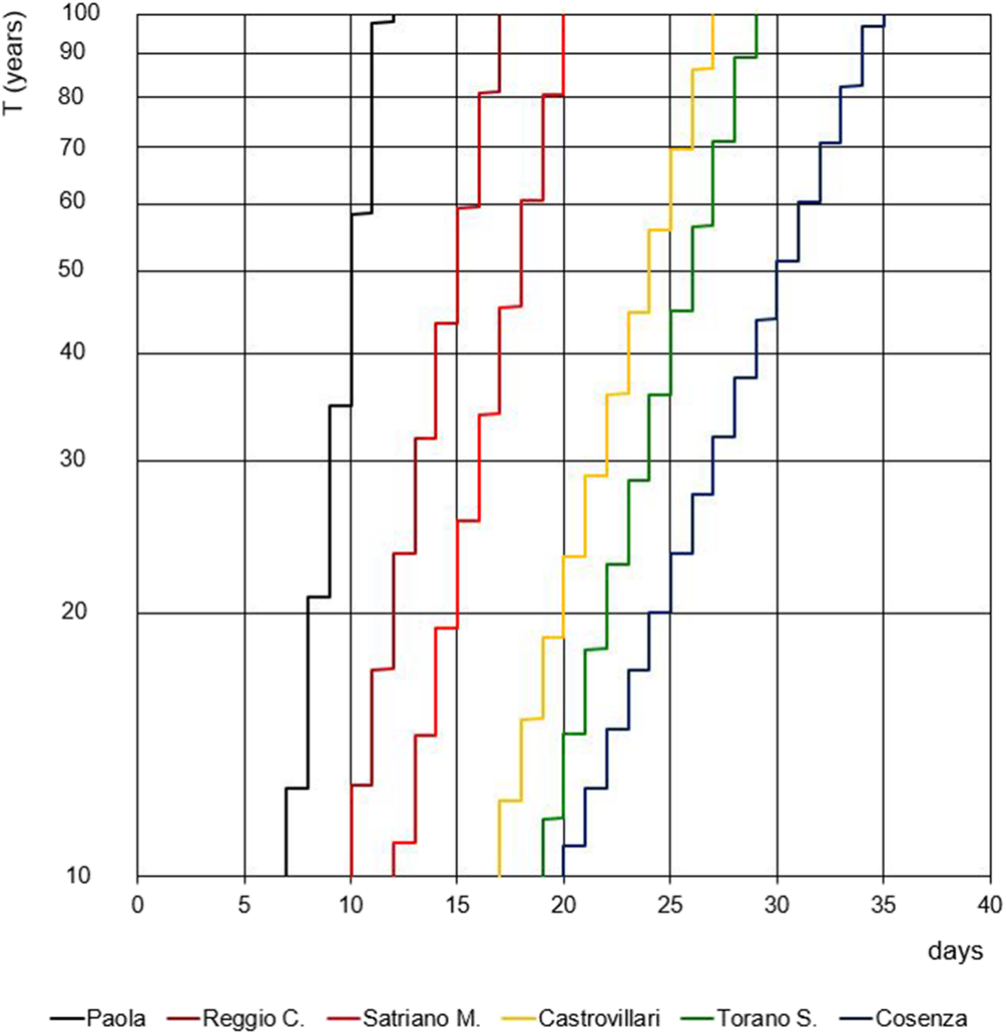
Comparison among annual maximum values of the number of consecutive days with maximum daily value of Humidex greater than 35 °C for all the stations.

Figure 8 is similar to Fig. 7 but the return periods have been estimated for all the stations and only for a Humidex threshold value equal to 35. Results show that, for their behaviour, the six stations can be divided into two groups: the first one includes the inner stations (Cosenza, Torano Scalo and Castrovillari), while the second one contains the stations near to the sea (Satriano Marina, Reggio Calabria and Paola), thus evidencing the possible influence of sea proximity. As an example, the occurrence of sequence of consecutive 20 days has a return period ranging from 10 years (Cosenza) to about 20 years (Castrovillari). Conversely, for the stations nearer to the sea this occurrence has return periods much higher than 100 years.

**Figure 9:**
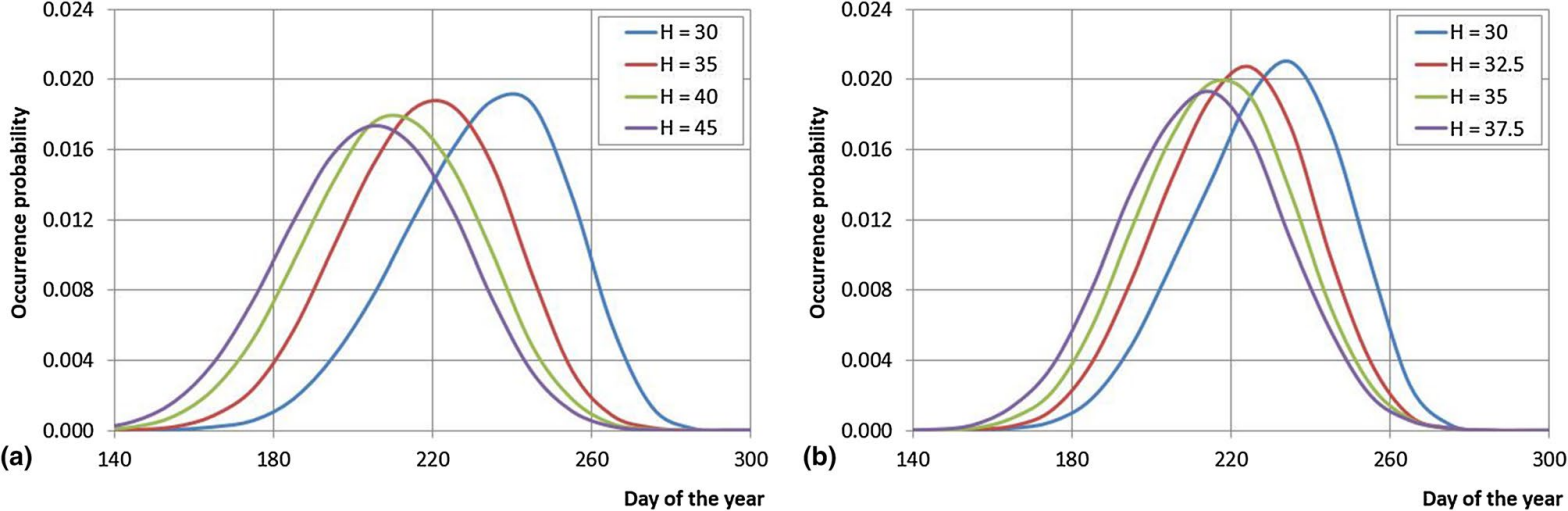
Occurrence distribution of the starting day of the sequences of consecutive days with maximum length of daily maximum Humidex greater than specified thresholds for the Cosenza and Satriano Marina stations.

It is interesting to estimate in which day of the year the highest occurrence probability to start a sequence of consecutive days with the daily maximum Humidex value higher than a prefixed threshold occurs. As an example, for the Cosenza and Satriano Marina stations (Fig. 9), results show that the lower the Humidex threshold is, later in the year the highest value of probability is registered. Moreover, the diagrams show also a tendency to a decrease of the highest probability: the higher the Humidex threshold is, the lower the occurrence probability. In fact, for the Cosenza station the highest probabilities during a year occur from the 205th (Humidex = 45; P_max_ = 0.017) to the 235th day (Humidex = 30; P_max_ = 0.019) while for the Satriano Marina station little higher probabilities than the Cosenza station have been observed, e.g. for Humidex = 30 the highest probability is 0.021.

**Figure 10:**
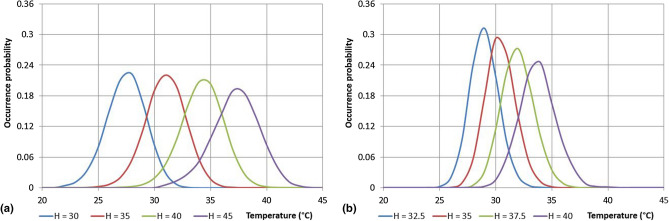
Distribution of the temperature for assigned values of the daily maximum Humidex for the Cosenza and Paola stations.

In order to detect the influence of the temperature value in the evaluation of the Humidex, it is interesting to analyse the occurrence probability of temperatures for fixed values of the maximum daily Humidex. As an example, for the Cosenza and the Paola stations, the results of this analysis show a tendency to a decrease of the maximum probabilities when the Humidex and temperature values increase (Fig. 10). In particular, for Cosenza, with a Humidex value of 35 the highest probability (0.220) is reached with a temperature of 31°, while with a Humidex equal to 40, the highest probability is lower (0.208) and corresponds to a temperature of 34°. Similarly, for the Paola station, maximum probability values of 0.291 (T = 30°) and of 0.245 (T = 34°) have been observed for Humidex equal to 35 and 40, respectively.

**Figure 11:**
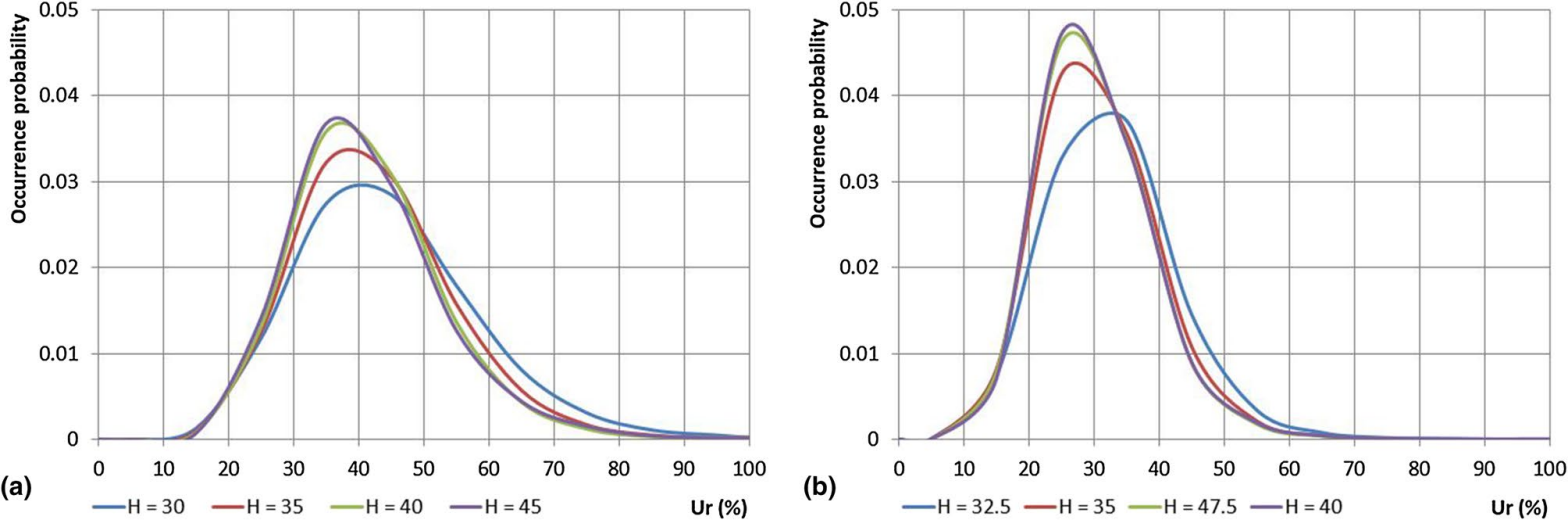
Distribution of the relative humidity for assigned values of the daily maximum Humidex for the Cosenza and Castrovillari stations.

Figure 11 shows diagrams analogous to those of Fig. 10, but referred to *Ur*, for the Cosenza and Castrovillari stations. Differently from the results obtained for the temperature, the curves are very close each other with the highest probability values falling within a short range of *Ur* values. Moreover, an opposite behaviour with respect to the temperature has been observed, with the maximum values of the probabilities that tend to fall when Humidex decreases. Specifically, for the Cosenza station, the highest probabilities range between 0.037 (Humidex = 45) for *Ur* = 35% to 0.029 (Humidex = 30) for *Ur* = 45%. For the Castrovillari station, higher maximum probabilities values and lower corresponding *Ur* values than the Cosenza stations have been observed: these data vary from 0.047 (Humidex = 40) for *Ur* = 25% to 0.037 (Humidex = 32.5) for *Ur* = 35%.

**Figure 12:**
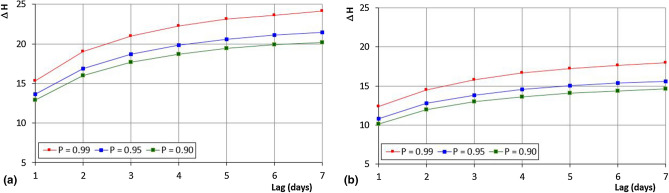
Quantiles of the annual maximum rises of the daily maximum Humidex for the Cosenza and Reggio Calabria stations.

A significant analysis can be performed to evaluate the excursion of the maximum daily Humidex values from one day to another. Figure 12 shows the maximum yearly increase of this excursion in 1–7 consecutive days for different probability values (90%, 95%, and 99%), for the Cosenza and Reggio Calabria stations. For Cosenza, this increase of Humidex values ranges between 12.9 (P = 90%) and 15.3 (P = 99%) for a lag of 1 day and can reach values between 20.2 (90%) and 24.2 (99%) for a lag of 7 days. For Reggio Calabria, the increases are lower than the Cosenza station. In fact, for a lag equal to 1 day, the Humidex values can span from 10.1 (90%) to 12.7 (99%), and, for a lag of 7 days, can range between 14.6 (90%) and 18 (99%).

**Figure 13:**
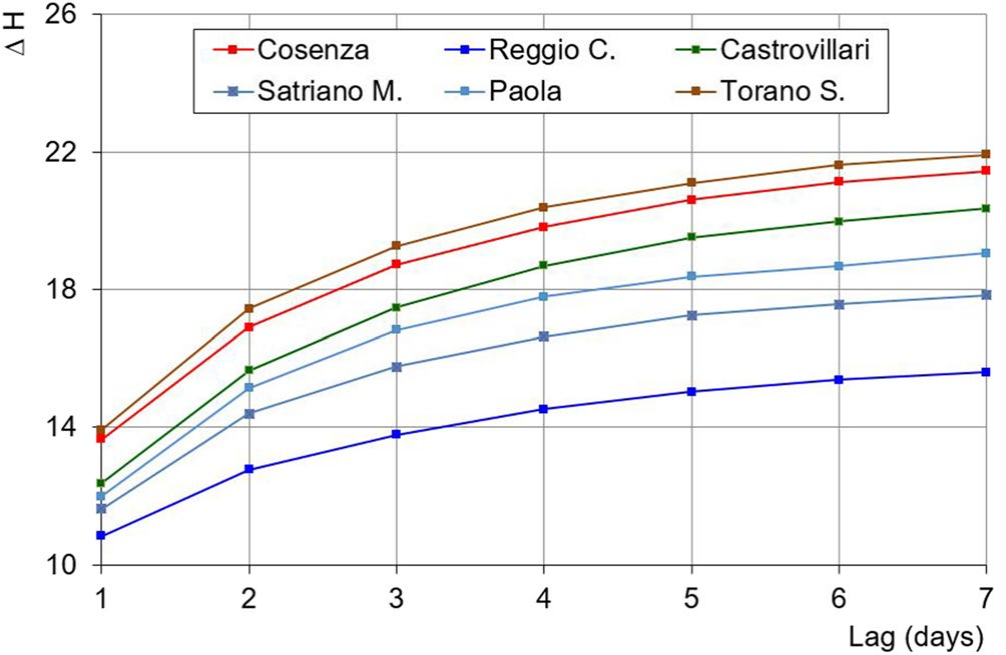
Comparison of the 95%-percentile of the annual maximum rises of the daily maximum Humidex for all the stations.

Similarly, in Fig. 13 a comparison of the maximum yearly Humidex increases for lags 1–7 consecutive days is shown, with reference to the 95% probability value and for all the stations. The curves of the Cosenza and Torano Scalo stations are very close and present the highest values of rise. Conversely, the Reggio Calabria station shows the lowest increases. In particular, Cosenza and Torano Scalo evidenced a ΔH of about 14 in just one day, reaching an increase value of about 22 in 7 consecutive days. On the contrary, for Reggio Calabria, ΔH values of about 11 and 14 have been obtained for a lag of 1 and 7 days, respectively.

**Figure 14:**
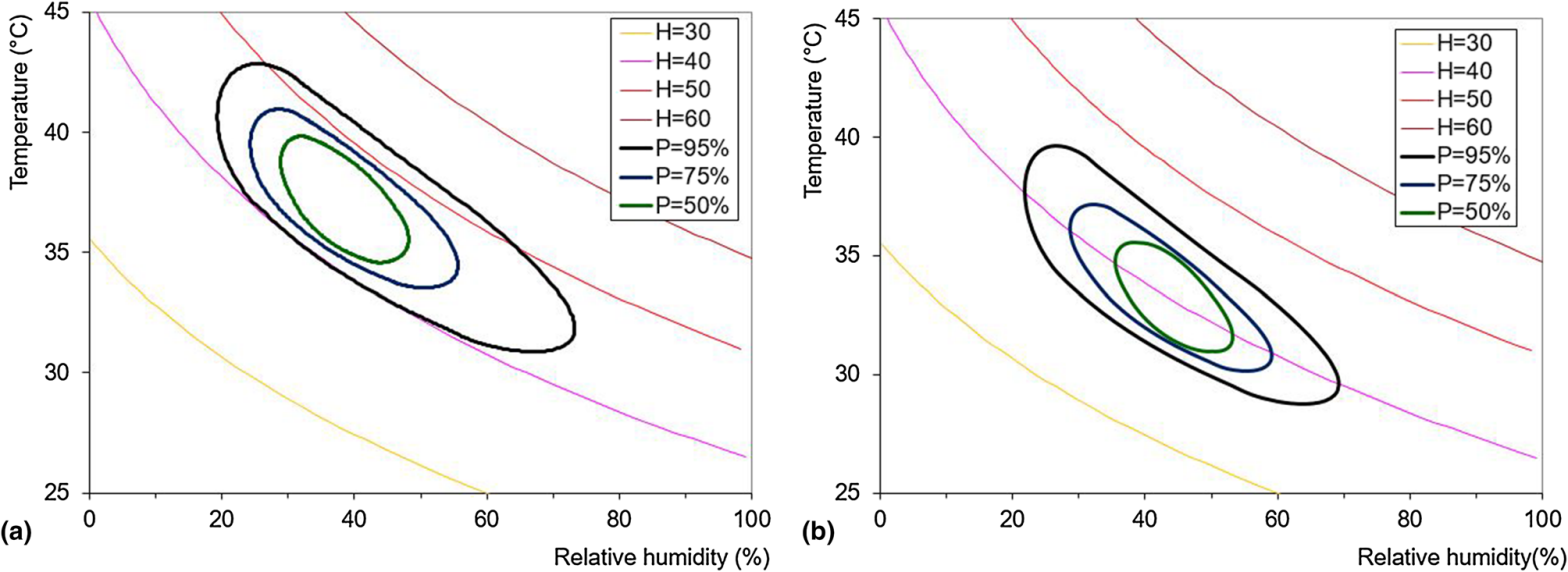
Behaviour of the maximum yearly Humidex values with varying temperature and relative humidity for the Cosenza and Reggio Calabria stations.

In order to evidence the combined influence of the variables *T* and *Ur* on the Humidex behaviour, the contour lines corresponding to different values of probability of the maximum yearly values of Humidex have been evaluated through the synthetic series. Figure 14 shows the results for three different probability values, i.e. 50%, 75%, and 95%, for the Cosenza and Reggio Calabria stations. For the Cosenza station the greatest part of the maximum yearly Humidex values ranges from 40 to 50, while for Reggio Calabria the curves are very close to theoretical 40-curve and ranges from about 38 and 45, thus evidencing higher probabilities of reaching serious danger conditions for Cosenza.

**Figure 15:**
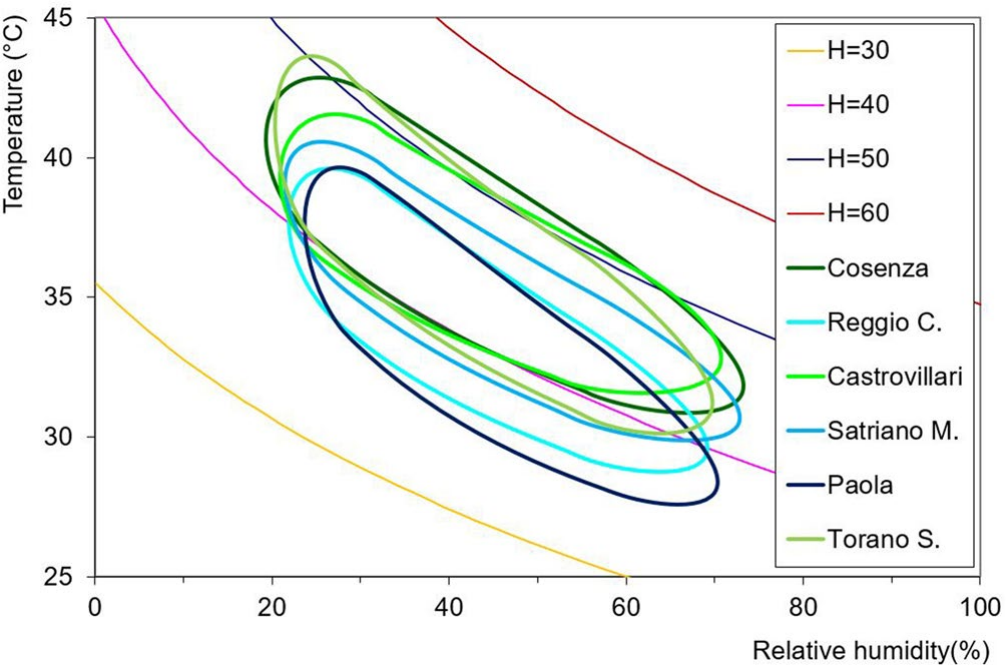
Comparison of the behaviour of the maximum yearly Humidex values for varying temperature and relative humidity for all the stations.

With reference only to the 95% probability value, the different behaviour of the maximum yearly Humidex values among all the stations is shown in Fig. 15. As a result, a marked difference has been detected between the stations nearer to the sea (Reggio Calabria, Paola, Satriano Marina) and the inner stations (Cosenza, Castrovillari, Torano Scalo), confirming the results shown in Fig. 13.

## Discussion and conclusions

Human-perceived equivalent temperature under extremely warm periods depends on the humidity conditions. Specifically, the relative humidity can compromise the body’s evaporative cooling mechanism, thus inducing a lower degree of wellness^43^. The occurrence of high value of an index such as the Humidex, which is based on temperature and humidity, is thus paramount for its impact on human health^44–47^. In fact, extreme heat conditions are characterized by values of Humidex beyond specified thresholds, which also take into account peculiar humidity conditions increasing the impact of temperature on people’s health^48^.

Usually, the quality of the time series of temperature and relative humidity is not good enough for statistical purposes, presenting missing values or too low years of observation. To overcome this problem, a stochastic model has been proposed and applied to six stations of Calabria, an Italian region often coping with summer periods characterized by very high temperature^41^. The model required the use of *FARIMA* processes to describe the correlative structures of temperature and relative humidity series, once duly deseasonalized and normalized. The goodness of the stochastic model has been assessed from the comparison of extreme observed Humidex values to the simulated Humidex series obtained by synthetic generation through a Monte Carlo procedure.

Focusing on the results provided by the application of the stochastic model, the maximum yearly values of consecutive days with maximum daily Humidex value greater than prefixed thresholds, for a fixed return period, shorten for increasing threshold values. In this way, a different behaviour has been recognized between inner stations and stations located near the coast. In fact, return periods corresponding to the same sequences of consecutive days with prefixed Humidex values are much higher for the coastal stations than for the interior ones. This evidences the possible influence of sea proximity, which appears to relieve uncomfortable conditions. This result confirms the outcomes of Cannistraro et al.^49^, which revealed better comfort conditions in coastal cities subject to constant ventilation than in inner areas, using different approach and index and comparing the data of various stations.

Moreover, the statistical investigation of the synthetic series evidenced the different influence of temperature and relative humidity on the Humidex behaviour. In fact, confirming what is widely stated in the literature^50^, the analyses show that the discomfort conditions are significantly related to air temperature, while the impacts of humidity are of less importance. More specifically, our results also confirm that the relative humidity contributes to the most dangerous discomfort conditions only within a narrow range of percentile values, due to a lower influence of relative humidity with higher temperature values^51^.

The consequences of the analyses confirm the importance of taking account of the Humidex, in order to detect promptly the occurrence of potential, unusual heat-related discomfort conditions. In other terms, the results obtained for specific communities could provide help to local health agencies in inferring about the possible occurrence of discomfort conditions.
